# Passive administration of monoclonal antibodies to Anthrolysin O prolong survival in mice lethally infected with *Bacillus anthracis*

**DOI:** 10.1186/1471-2180-8-159

**Published:** 2008-09-23

**Authors:** Antonio Nakouzi, Johanna Rivera, Richard F Rest, Arturo Casadevall

**Affiliations:** 1 Department of Microbiology and Immunology, Albert Einstein College of Medicine, 1300 Morris Park Avenue, Bronx, New York 10461, USA; 2 Department of Medicine (Division of Infectious Diseases), Albert Einstein College of Medicine, 1300 Morris Park Avenue, Bronx, New York 10461, USA; 3 Department of Microbiology and Immunology, Drexel University College of Medicine, 2900 Queen Lane, Philadelphia, PA 19129, USA

## Abstract

**Background:**

*Bacillus anthracis *has two major virulence factors: a tripartite toxin that produces lethal and edema toxins and a polyglutamic acid capsule. A recent report suggested that a toxin belonging to the cholesterol dependant cytolysin (CDC) family, anthrolysin O (ALO) was a new virulence factor for *B. anthracis *but subsequent studies have questioned its relevance in pathogenesis. In this study, we examined the immunogenicity of recombinant anthrolysin O (rALO) in mice.

**Results:**

BALB/c mice immunized with rALO and boosted after two weeks, produce sera with strong Ab responses with a predominance of IgG1 and IgG2a. Five hybridomas to rALO were recovered representing the IgM, IgG1, and IgG2b isotypes. Passive administration of 3 of the five monoclonal antibodies (mAbs) to rALO prior to infection with lethal intravenous (i.v.) *B. anthracis *Sterne strain infection in mice was associated with enhanced average survival and a greater likelihood of surviving infection. A combination of two mAbs to ALO was more effective than either mAb separately. One mAb (64F8) slowed the toxicity of rALO for J774.16 macrophage-like cells.

**Conclusion:**

Our results suggest that ALO contributes to the virulence of *B. anthracis *Sterne strain in this infection model and that Ab response to ALO may contribute to protection in certain circumstances.

## Background

*Bacillus anthracis *is gram-positive bacterium that is the causative agent of anthrax, a fulminant disease of grazing animals. In recent years, *B. anthracis *has emerged as a major biological weapon as evidenced by the casualties and disruption caused in 2001 by distribution of the spores through the U.S. Mail. Consequently, there is great interest in understanding its pathogenesis as well as in the development of countermeasures to prevent and/or treat disease. *B. anthracis *has two well-studied virulence factors: a polyglutamic acid capsule and a tripartite toxin that comprises lethal toxin (LT) and edema toxin (ET). Ab-mediated immunity neutralizes these toxins as evidence for reduced susceptibility after immunization with anthrax toxin and/or capsular antigens.

In addition to LT and ET, *B. anthracis *expresses several other toxins including a cholesterol-dependent cytolysin (CDC) known as anthrolysin O (ALO) [1]. The CDC class of toxins includes listeriolysin O, perfringolysin O, and streptolysin O, in *Listeria*, *Clostridia*, and *Streptococcus *spp, respectively. Given that CDCs are important virulence factors for Gram positive bacteria, ALO was initially considered to be a potential virulence factor [1] and a role in pathogenesis was envisioned from the observation that it interacted with Toll-like receptor 4 [2]. Furthermore, *B. anthracis *ALO kills neutrophils and macrophages *in vitro *[3]. However, a comparison of ALO-deficient and wild-type strains in an inhalation anthrax model revealed no difference in virulence, suggesting that this toxin was not essential for virulence [4]. Consistent with this observation, immunization with ALO protects mice against the toxin but not infection with *B. anthracis *[5]. Hence, the current view is that ALO is a potent cytotoxin *in vitro *that has not been demonstrated to have a significant role in virulence.

In this study we revisited the role of ALO in virulence by generating mAbs to recombinant ALO and employing them in passive protection experiments. We report the generation of five mAbs that differ in their efficacy for toxin neutralization *in vitro *and their ability to prolong survival in mice infected with *B. anthracis*. These results are interpreted as implying that ALO contributes to the pathogenesis of anthrax, at least in experimental murine intravenous infection.

## Results

### Immunogenicity of rALO

**Figure 1:**
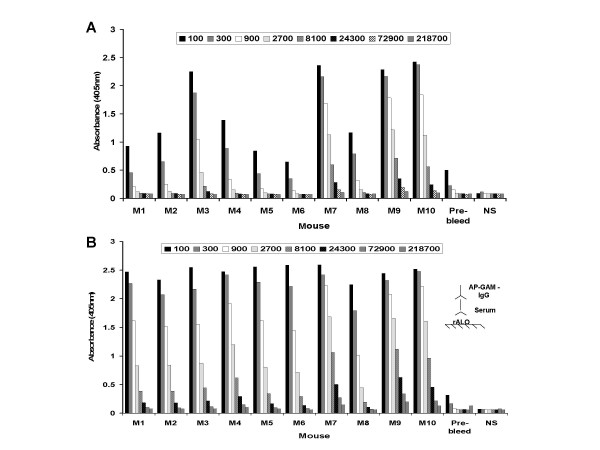
**A. Antibody (Ab) response of mice immunized with recombinant anthrolysin (rALO) after initial immunization at day 14 as determined by ELISA.** Immunization was done with complete Freund's adjuvant. Serum dilution is represented by different fill patterns. **B. **Ab response after mice were boosted with same amount toxin in incomplete Freund's adjuvant measured at day 28. Individual mouse are denoted by M and numbers 1 to 10. Mice 1–5 received 1 μg of rALO and mice 6–10 received 10 μg of rALO. Pre-bleed is serum from mouse 10 before immunization and was used as control in panels A and B. Alkaline phosphatase conjugated goat anti-mouse IgG (AP-GAM-IgG) alone was used a negative control (NS: no serum) for A and B. Inset, ELISA configuration for both figures whereby rALO is absorbed on polystyrene plates and the presence of specific Ab to rALO is detected with AP-GAM-IgG.

**Figure 2:**
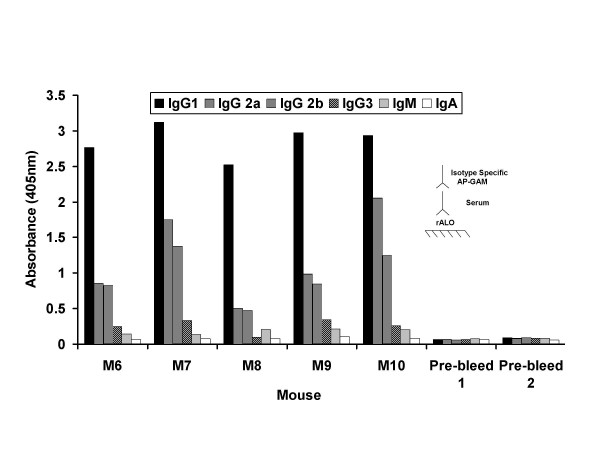
**Isotype composition of Ab response of mice immunized with rALO.** Serum from M6–M10 was analyzed for isotype Ab response to rALO. Serum was diluted 1:100. The different isotypes were represented by different fill patterns. Inset denotes ELISA configuration whereby rALO is absorbed on polystyrene plates and the presence of specific Ab to rALO is detected with AP-GAM-IgG. Pre-bleed 1 and 2 are from M9 and M10 before immunization with rALO.

rALO was emulsified in complete Freund's adjuvant and five BALB/c mice were injected with either 1 or 10 μg/mouse. All mice responded to immunization with a serum Ab response to rALO although there were significant individual differences among mice in the magnitude of the response (Figure 1). At two weeks, mice were boosted with the same amount of rALO in incomplete Freund's adjuvant. The anti-rALO response was boosted with subsequent immunization. The Ab response was composed mainly of IgG1 and IgG2a, with significantly smaller amounts of IgG2b and IgG3, and very little IgA and IgM (Figure 2). The mouse with the highest anti-ALO Ab titer (mouse M9, Figure 1B) was selected for spleen harvest and hybridoma generation. Five mAbs were recovered; three IgMs (53C2, 62F7 and 80C9), one IgG1 (64F8) and one IgG2b (16G2).

### Specificity studies

**Figure 3:**
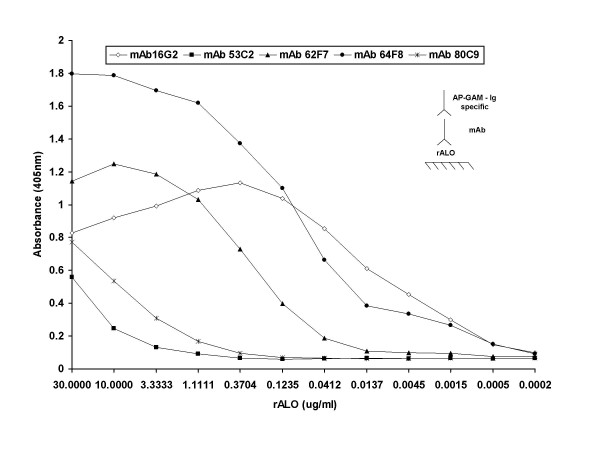
**MAb binding to rALO by ELISA.** MAbs isolated were examined for their binding abilities to rALO. ELISA configuration includes absorbing rALO onto polystyrene plates and specific Ab binding to rALO is detected with AP-GAM-IgG. Diagram illustrates the ELISA configuration.

**Figure 4:**
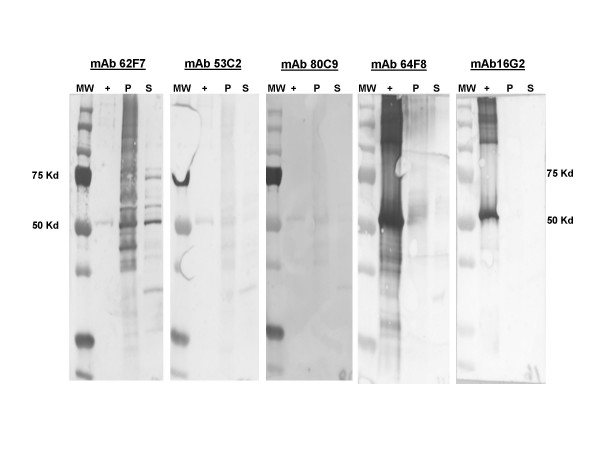
**Immunoblot analysis of mAb binding to rALO and *B. anthracis *Sterne strain.** rALO, culture supernatant, and lysed bacterial cells were solubilized in Laemmli sample buffer, run under denaturing conditions and were separated on a 10% SDS-PAGE. The blots were incubated with rALO specific mAbs and isotype specific GAM secondary Ab conjugated to HRP. MW, molecular weight marker; +, rALO; P, lysed bacterial cells; S, culture supernatant. The expected molecular weight of rALO is approximately 53 Kd.

The mAbs generated were tested for their ability to bind rALO by ELISA and immunoblot (Figures 3 and 4). Three of the mAbs (16G2, 62F7, and 64F8) manifested significantly higher levels of reactivity with rALO by ELISA than the other two (53C2 and 80C9) (Figure 3). To obtain some information on the specificity of these mAbs competition ELISAs were carried out with different pairwise combinations of Abs. mAb 80C9 (IgM) and mAb 64F8 (IgG1); mAb 53C2 (IgM) and mAb 16G2 (IgG2b); mAb 62F7 (IgM) and mAb 64F8 (IgG1); mAb 80C9 (IgM) and Mab 16G2(IgG2b); mAb 53C2(IgM) and mAb 64F8(IgG1); mAb 62F7(IgM)and 16G2(IgG2b). None of the mAbs inhibited the binding of any other mAb, implying that each of these pairs binds to a different epitope (data not shown). Immunoblot analysis revealed that all five mAbs bound to rALO and to ALO from lysed *B. anthracis *cells and their culture supernatants (Fig 3). However, the intensity of the mAb reactivity with rALO by immunoblot varied with mAbs 16G2 and 64F8 producing significantly stronger signals than the rest of the mAbs. The intensity of the reactivity with ALO produced by the Sterne strain was strongly recognized by mAb 62F7 and weakly by the rest of the mAbs.

### *In vitro* neutralizing activity

Given that ALO is cytotoxic *in vitro*, we examined whether the various anti-rALO mAbs could reduce killing of macrophages by rALO *in vitro*. These experiments necessarily involved measuring toxicity after relatively short incubation times because of the rapid lytic action of rALO. MAb antitoxic activity was assessed by an *in vitro *assay wherein cell viability was measured by trypan blue staining. Only mAb 64F8 manifested *in vitro *toxin neutralizing activity (50% cell viability compared to the other mAbs and irrelevant Ab).

### Survival studies

**Figure 5:**
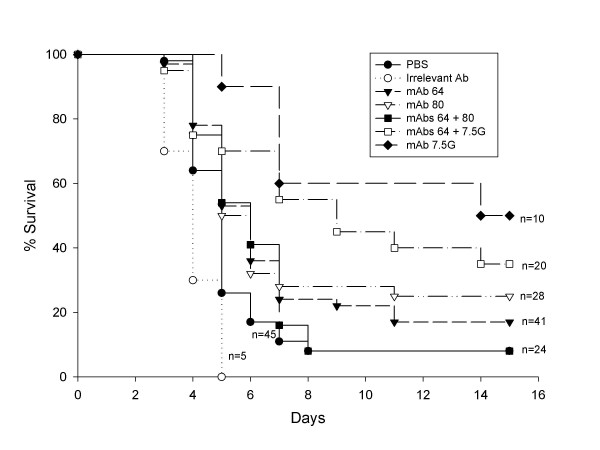
**Survival analysis of A/JCr mice infected with *B. anthracis *Sterne strain.** Mice were given 100 μg mAb intraperitoneally 2–3 h prior to infection and infected intravenously with 10^4 ^bacterial cells. Mice were monitored daily for morbidity. The data shown represents pooling of six independent experiments using the same conditions.

**Figure 6:**
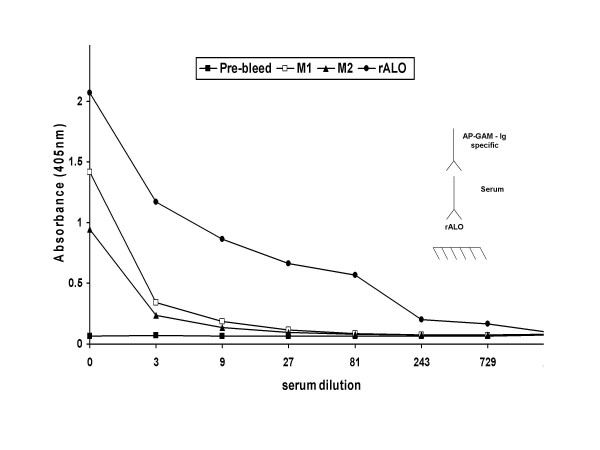
**Reactivity of serum from moribund mice lethally infected with *B. anthracis *Sterne strain.** These mice were from control groups that did not receive passive mAb to rALO. Pre-bleed serum was collect from M1 and 2 before infection and was used at a dilution of 1:50. rALO (1 μg/ml) was used as the positive control. Ab titers were measured at day 4 of post-infection. Inset denotes ELISA configuration. M1 and 2 denotes mouse 1 and 2.

To evaluate the protective efficacy of the anti-rALO mAbs *in vivo*, we administered mAbs i.p. 2 h to 3 h prior to intravenous infection with *B. anthracis*. A total of 6 independent passive Ab experiments were performed. MAbs 16G2 and 62F7 showed no protection in any of the survival studies while mAbs 53C2 (Table 1), 64F8 and 80C9 revealed a moderate level of protection (Figure 5). We selected a dose of 0.1 mg/mouse after noting that larger doses were either not more effective or were associated with reduced protection in prozone-like effects (Table 1). In the majority of experiments, mAbs 64F8 and 80C9 were associated with modest but statistically significant prolongations in survival relative to groups receiving an irrelevant mAb or PBS. The proportion of mice surviving *B. anthracis *challenge was 20% (32/128) in mice receiving anti-ALO mAbs compared to 8% (4/50) in control groups receiving either PBS or an irrelevant Ab surrogate (P = 0.049). The combination of mAbs 64F8 and 80C9 was more effective than either mAb alone in prolonging survival (Table 1). The protection observed with passive administration of mAbs to ALO was lower than observed for a previously described neutralizing mAb to PA (Figure 5). Passive administration of mAbs to PA (mAb 7.5G) and ALO (mAb 64F8) revealed that their effect on survival was not additive or synergistic in this system (Fig 5). Serum from moribund animals manifested reactivity with ALO indicating that the enzyme is made during the course of infection, that it is immunogenic, and that a brisk Ab response is apparent by day 4 of infection (Figure 6).

**Table 1:** Survival analysis of A/Jcr mice treated with mAb prior to i.v. infection with *B. anthracis *Sterne strain 34F2

Exp.	Group Condition	mAb Dose (mg/mouse)	n	Mean survival (Days)	Median Survival (Days)	Survive^1^	*P *value^2^
I	PBS		8	4.13	4	0	
	64F8 (IgG1)	0.25	8	5.75	4	1	.14
	64F8	0.5	8	4.88	4	0	.10
	64F8	1.0	8	7.25	5	2	.02
	80C9 (IgM)	0.25	8	8.25	4	3	.05
	80C9	0.5	8	5.88	4	1	.09
	80C9	1.0	8	4.50	4	0	.20
	64F8 + 80C9	0.5 + 0.5	4	8.0	6	1	0.002
							
II	PBS		10	5.40	5	0	
	53C2 (IgM)	0.1	10	8.40	6	1	0.02
							
III	PBS		10	3.90	4	0	
	64F8 + 16G2 (IgG1+IgG2b)	0.1 + 0.1	10	4.60	4	0	0.023
	64F8 + 53C2 (IgG1+gM)	0.1 + 0.1	10	4.20	4	1	0.44
	64F8 + 80C9 (IgG1+IgM)	0.1 + 0.1	10	4.70	4	0	0.09
							
IV	PBS		10	5.20	5	0	
	64F8 (IgM)	0.1	10	6.90	6	1	0.06
	80C9 (IgM)	0.1	10	8.10	6	2	0.03
	64F8 + 80C9	0.1 + 0.1	10	7.30	7	1	0.01

^1^Number of long-term survivors

^2 ^P value relative to PBS by log rank analysis

## Discussion

The role of ALO in *B. anthracis *pathogenesis is uncertain because ALO-deleted strains have not manifested reduced virulence relative to ALO competent strains. Furthermore, a vaccination study with an ALO toxoid vaccine revealed that immunization protected against toxin challenge but not *B. anthracis *infection [5]. In this study, we have revisited the role of ALO in *B. anthracis *pathogenesis by making mAbs to rALO and assessing their ability to modify the course of lethal infection in mice. Since immune responses often target and negate the function of virulence factors such as toxins and capsules, the ability to demonstrate that active and/or passive immunity to a particular microbial component can mediate protection is a time-honored method for establishing the importance of that component in virulence. Using standard hybridoma technology, we generated five mAbs to ALO. We demonstrated that passive administration of three of these mAbs before infection prolonged average mouse survival after lethal *B. anthracis *infection and significantly increased individual mouse survival after inoculation. The average increase in survival time observed after passive administration of anti-ALO mAbs was shorter than that observed after administration of neutralizing mAb to PA. Based on these results we conclude that ALO contributes to the overall virulence phenotype of *B. anthracis *Sterne strain with the caveat that the relative contribution of this toxin is probably significantly less than that of other well-established virulence factors such as LT, ET and capsule, possibly because these bacteria expresses redundant phospholipases [4]. Since the overall virulence phenotype of a pathogenic microbe is a function of the combination of its virulence attributes, it is possible that ALO makes a disproportionately greater contribution to virulence for the *B. anthracis *Sterne strain because this strain lacks a capsule. This conclusion does not negate the fact that no difference in virulence was found for ALO-deficient and – sufficient *B. anthracis *strains [4], since the contribution of ALO to virulence may not be sufficient to manifest itself in that comparison. Determining the relative contribution of ALO to the overall virulence *B. anthracis *virulence phenotype cannot be estimated from this data, but such information may be obtained by regression analysis of multiple strains that differ in virulence factor expression [7,8].

Our results also shed some light on the difficulties associated with demonstrating a protective role for ALO toxoid immunization [5]. Of the five mAbs tested in this study, two were not protective indicating that ALO immunization elicited both protective and non-protective antibodies. In other systems non-protective Abs can interfere with the function of protective Abs [9,10]. In our passive protection experiments, we used a relatively large dose of Ab to obtain a modest survival effect. If *B. anthracis *is phagocytosed rapidly after i.v. infusion this large dose may reflect a requirement for high serum concentration to mediate intracellular effects as has been demonstrated for Ab efficacy against *Listeria monocytogenes *[11,12]. Another finding that may obscure differences between immunized and non-immunized hosts is that *B. anthracis *infection led to a rapid Ab response to ALO, thus diminishing the time window when there is a difference between immune and naïve hosts to the first few days of infection. It is noteworthy that moribund mice had measurable Ab to ALO implying that that the enzyme is expressed during infection and that an antibody response to this antigen is not sufficient to confer survival. Although the mechanism of antibody-mediated protection was not determined it is likely that it involved interference with the action of Anthrolysin. Consistent with this notion, one mAb exhibited transient neutralizing activity in vitro. The negative and/or modest effects toxin neutralizing effects may reflect the inadequacy of antibody-mediated neutralization given the fulminant nature of Anthrolysin-mediated cell lysis in vitro (eg a false-negative effect with this assay).

It is noteworthy that 3 of the 5 mAbs recovered were IgM despite the relatively scarcity of ALO-binding IgM in immune sera. Dissociation between serum isotype composition and recovered hybridomas is not unusual, and can reflect the fact that IgM half-life is in the order of hours while that of IgG is up to 8 days in mice. IgM hybridomas may also be preferentially selected during screening because this isotype has higher avidity.

The combination of two protective mAbs (64F8 and 80C9) was more effective than when the mAbs were administered singly. This observation implies that polyclonal preparations could be significantly more effective than single mAbs. Similar reports of mAb synergy have been reported in passive protection studies against pneumolysin [13] and other toxins [14,15]. The mechanism of Ab action *in vivo *is unclear. One of the three protective mAbs (mAb 64F8) delayed cell death for macrophages treated with ALO *in vitro*, suggesting that the mechanism of protection for this mAb could be toxin neutralization. For mAb 16G2, immunoblot binding studies revealed strong binding to rALO but no binding to *B. anthracis *expressing surface ALO. Hence, for this mAb the lack of protective efficacy could reflect an inability to bind to natively expressed *B. anthracis *ALO. We noted differences in the reactivity of mAbs with rALO and ALO from *B. anthracis *lysates. This observation implies antigenic differences between natively expressed *B. anthracis *and *E. coli *rALO that could reflect differences in conformation and/or structure between these proteins

## Conclusion

In summary, we report the generation of first mAbs to ALO and demonstrate that some confer protection in passive protection experiments against a non-encapsulated *B. anthracis *strain. This result suggests that eliciting Ab responses to ALO or providing antibodies passively could have clinical applications in prevention or therapy of anthrax. The finding that some mAbs prolong survival and that combinations of these mAbs are more effective than single mAbs suggests that it may be possible to identify more effective immunoglobulin preparations for use in prophylaxis and therapy. The observation that administration of Ab to ALO was associated with reduced mortality implies that ALO contributes to the virulence of *B. anthracis *infection, although the magnitude of the contribution ALO to the overall virulence phenotype remains to be established.

## Methods

### *Bacillus anthracis *strain 34F2 (Sterne strain) and rALO

*Bacillus anthracis *Sterne strain 34F2 (pXO1+, pXO2-) was obtained from Dr. Alex Hoffmaster at the Center for Disease Control (Atlanta, GA). Bacterial cultures were grown in brain heart infusion (BHI) broth (Difco, Detroit, Mich) at 37°C for 18 h while shaking. These conditions apply to all experiments. Recombinant anthrolysin (rALO) was expressed from *E. coli *as described [1].

### Mice and immunization

Female BALB/c and A/JCr mice (6–8 weeks old) were obtained from the National Cancer Institute (Bethesda, MD). Mice were immunized intraperitoneally (i.p.) with either 1 or 10 μg of rALO in complete Freund's adjuvant (Sigma, St. Louis, MO) and boosted after two weeks with 1 and 10 μg in incomplete Freund's adjuvant. The mouse with the highest anti-ALO serum titer was selected for generating hybridomas.

### ELISA

Enzyme-linked immunosorbant assay (ELISA) was used to determine serum Ab titers and mAb binding to rALO. Briefly, 1 μg/ml of rALO in phosphate buffered saline (PBS) was used to coat polystyrene microtiter plates. The plates were blocked with 1% bovine serum albumin in PBS (1%BSA/PBS). Microtiter plates were then incubated with immune sera or hybridoma supernatants. Ab binding was detected using isotype specific alkaline-phosphatase (AP) labeled goat anti-mouse (GAM) Ab (Southern Biotechnology, Birminham, AL). Plates were developed with *p*-nitrophenylphosphate (PNPP) substrate (Sigma, St. Louis, MO). All incubations were done at 37°C for 1 h. Absorbance was measured in a microplate reader at 405 nm (Labsystems Multiskan). Competition ELISAs were used to evaluate epitope specificity for the mAbs generated. Briefly, differing amounts of one of the mAbs was mixed with a constant amount of a second mAb of a different isotype. The mixture was added to rALO immobilized on a microtiter plate. After 1 h incubation at 37°C, the microplate was washed to remove excess unbound Ab. Ab binding was detected with isotype-specific AP labeled GAM Ab followed by a second incubation and wash. Detection was accomplished by the addition of PNPP substrate and measurement of absorbance at 405 nm.

### Generation of monoclonal antibodies

The mouse with the highest Ab titer was selected to generate Ab-producing hybridomas using previously established methods [6]. Briefly, NSO myeloma cells were used as fusion partners for the splenocytes from the immunized mouse, at a ratio of 4:1. Seven to ten days later, hybridoma supernatants from clones were screened by ELISA as described above for the presence of mAbs to rALO. Positive clones were selected and stabilized by cloning twice in soft agar. ELISA was used to determine the isotypes of the murine mAbs by using isotype-specific reagents.

### SDS-PAGE and Western blots

rALO, culture supernatant, and lysed bacterial cells were solubilized in Laemmli sample buffer and run under denaturing conditions. After boiling samples for 10 min, proteins were separated on a 10% SDS-PAGE. Proteins were visualized by staining for 1 h with GelCode stain (Pierce, Rockford, IL) and excess stain removed with deionized water. After samples were separated, they were transferred to a 0.20 μm nitrocellulose membrane and blocked with 3% powdered milk in PBS. The blots were incubated with the rALO specific mAbs, washed with 1% BSA/PBS and incubated with isotype specific GAM secondary Ab conjugated to HRP. Proteins were visualized by developing with a SuperSignal West Pico chemiluminescence kit (Pierce, Rockford, IL).

### Cell viability studies

Trypan blue solution was used to identify J774 macrophages that had been lysed after exposure to rALO. Cells (10^4^) were added to 96-well plate and immediately added 85 ng diluted in PBS of rALO. The 96-well plate was incubated for 30 min at 37°C and cell viability was visualized by microscopic examination.

### Survival studies

Six to eight week old A/JCr mice were injected i.p. with 0.1, 0.25, 0.5 or 1.0 mg of mAb 3 h prior to intravenous (i.v.) infection with a lethal dose (10^4 ^bacterial cells) of *B. anthracis*. Mice were monitored daily for mortality and morbidity and deaths recorded.

### Statistical analysis

Survival analysis was done by log rank censoring long term survivors. The chi square statistic (p < 0.05) was used to determine the significance between groups.

## Authors' contributions

AN carried out the generation of monoclonal antibodies, serological studies. JR participated in the survival studies. RFR contributed in the generation of the recombinant anthrolysin O. AC conceived the study and participated in the design and coordination of the manuscript. All authors read and approved the final manuscript.
